# Comparative analysis of health status and health service utilization patterns among rural and urban elderly populations in Hungary: a study on the challenges of unhealthy aging

**DOI:** 10.1007/s11357-023-00926-y

**Published:** 2023-10-06

**Authors:** Nora Kovacs, Peter Piko, Attila Juhasz, Csilla Nagy, Beatrix Oroszi, Zoltan Ungvari, Roza Adany

**Affiliations:** 1 Department of Public Health and Epidemiology, Faculty of Medicine, University of Debrecen, Debrecen, Hungary; 2 ELKH-DE Public Health Research Group, Department of Public Health and Epidemiology, Faculty of Medicine, University of Debrecen, Debrecen, Hungary; 3 Center for Epidemiology and Surveillance, National Laboratory for Health Security, Semmelweis University, Budapest, Hungary; 4 Vascular Cognitive Impairment and Neurodegeneration Program, Oklahoma Center for Geroscience and Healthy Brain Aging, Department of Biochemistry and Molecular Biology, University of Oklahoma Health Sciences Center, Oklahoma City, OK USA; 5 Department of Health Promotion Sciences, College of Public Health, University of Oklahoma Health Sciences Center, Oklahoma City, OK USA; 6 International Training Program in Geroscience, Doctoral School of Basic and Translational Medicine, Departments of Public Health and Translational Medicine, Semmelweis University, Budapest, Hungary; 7 The Peggy and Charles Stephenson Cancer Center, University of Oklahoma Health Sciences Center, Oklahoma City, OK USA; 8 Department of Public Health, Semmelweis University, Budapest, Hungary

**Keywords:** Population ageing, Preventive services, Place of residence, Central and Eastern Europe (CEE), Older adults

## Abstract

The demographic transition poses a significant challenge for health systems, especially in Central and Eastern European (CEE) countries, where the healthcare needs of aging populations are on the rise. This study aimed to describe and compare the health status and utilization of health services among the elderly residing in urban and rural areas of the most deprived region in Hungary. A comprehensive health survey was conducted in 2022, involving a randomly selected sample of 443 older adults (≥ 65 years) in Northeast Hungary. Multivariable logistic regression models adjusting for age, sex, education, financial status, chronic diseases, and activity limitations were used to investigate the association between type of residence and health service use. Among the study participants, 62.3% were female, 38.3% attained primary education, 12.5% reported a bad or very bad financial situation and 52.6% lived in urban areas. Overall, 24% of the elderly rated their health as very good or good (27.8% in urban and 19.7% in rural areas), while 57.8% (52.6% and 63.5% in urban and rural areas) reported limitations in daily activities. Compared to urban residents, rural residents reported lower rates of dentist visits (*p* = 0.006), specialist visits (*p* = 0.028), faecal occult blood testing (*p* < 0.001), colorectal cancer screening with colonoscopy (*p* = 0.014), and breast cancer screening (*p* = 0.035), and a higher rate of blood pressure measurement (*p* = 0.042). Multivariable models indicated that urban residence was positively associated with faecal occult blood testing (OR = 2.32, *p* = 0.014), but negatively associated with blood pressure (OR = 0.42, *p* = 0.017) and blood glucose measurements (OR = 0.48, *p* = 0.009). These findings highlight the influence of residence on health service utilization among older adults in Hungary. Further comprehensive studies are needed to better understand the health needs of the elderly population and to develop policies aimed at promoting healthy aging in CEE countries.

**Supplementary Information:**

The online version contains supplementary material available at 10.1007/s11357-023-00926-y.

## Introduction

The global demographic transition towards an aging population presents substantial challenges for health systems worldwide [1]. As older age is associated with an increased prevalence of non-communicable diseases and chronic conditions, there is a growing demand for care, resources, and health technologies to address these health needs [2]. However, the progress of population aging and demographic change varies across regions, with Europe experiencing an advanced stage of this transition [3]. In the European Union (EU), the proportion of those aged 65 years and over is projected to rise from 19% (in 2016) to 29% of the population in 2070 in EU27 [4]. Consequently, the EU is likely to face a considerable burden associated with age-related conditions and a surge in demand for medical and social services.

Central and Eastern European (CEE) countries, including Hungary, have undergone significant changes in their health and social care systems during this transition and are confronted with challenges related to the growing health needs of their aging populations [5]. Compared to other member states of the EU, CEE countries, including Hungary, demonstrate lower health status indicators, characterized by higher rates of avoidable mortality attributed to conditions such as ischemic heart disease and treatable cancers [6]. Previous studies have demonstrated that older individuals in CEE countries report poorer self-reported health, higher prevalence of long-term illnesses, chronic diseases, and obesity [7]. Additionally, studies in countries such as Poland, Croatia, and Romania have shown that the health status of older adults in these regions is generally poorer compared to their counterparts in Western Europe [8, 9]. For instance, elderly individuals in Romania reported worse mental health, impaired social functioning, and greater difficulties in daily activities [9].

Hungary, among both EU and OECD countries, exhibits an unfavorable position in terms of health status, indicating an unhealthy aging population. Although there has been a decline in treatable mortality across all OECD countries in recent years, Hungary still experiences rates 1.8 times higher (131/100,000 population) than the OECD average in 2019 [10]. Similarly, preventable mortality rates remain nearly twice as high (243/100,000) in Hungary compared to the OECD average [10]. The life expectancy at birth in Hungary in 2021 was 74.5 years, which is 5.6 years less than the EU average [11]. Furthermore, Hungary recorded a significant number of potential years of life lost due to premature death (before the age of 70), with rates of 6,884 years (9,527/100,000 for men, 4,496/100,000 for women) per 100,000 inhabitants in 2019, ranking it 5th highest among EU27 countries [12].

With nearly 2 million people aged 65 or above, accounting for 20.5% of the total population in 2022, Hungary's aging population is growing, and it is projected to reach over 26% by 2050 [13]. However, Hungary, along with other CEE countries such as Poland, Estonia, and Slovenia, ranks at the bottom of the Aging Society Index, which was developed based on an evidence-based model incorporating the five major components for the successful ageing of a society (including productivity and engagement, well-being, equity, cohesion, and security), indicating a greater need for improvement in adapting to the demographic transition [14]. The dynamics of the aging process has progressed in Hungary in recent decades, as evidenced by the Aging Index, which measures the ratio of the population aged 65 and older relative to the population aged 14 and younger. The index has increased from 99.9 in 2005 to 141.1 in 2022 [13], emphasizing the pressing need for studies focusing on the healthcare demands of the older population. The Northeastern region of Hungary, known for its high socioeconomic deprivation, exhibits lower life expectancy [15] and higher premature mortality rates [16, 17]. Given the aging population and the associated rise in morbidity, it is crucial to gain a better understanding of the healthcare needs of the older population in this region.

Health disparities among the elderly are influenced by age, socioeconomic factors, and geographic location [18]. Older individuals experience higher prevalence of chronic diseases, physical impairments, and comorbidities, which necessitate increased utilization of ambulatory, inpatient, and chronic care services. Additionally, older people also frequently need medical care for other acute health problems, as well as guideline recommended preventive care services (including vaccination and screening for cardiometabolic conditions such as diabetes, hypertension, hypercholesterolemia, and certain cancers). However, the utilization of healthcare services among older adults can be influenced by factors such as access to services [19]. Although increased healthcare utilization is expected with age [20], there are often disparities in service provision between urban and rural areas, with rural populations facing limited access to healthcare services and professionals [21, 22]. Consequently, rural residents may experience delayed or inadequate care.

To address health inequalities and anticipate the demand for healthcare services among older populations, comprehensive data on the health status of older adults are required. However, limited data exists on various aspects of health and healthcare utilization among the elderly in CEE countries, particularly in relation to the type of residence. Therefore, this study aims to (1) describe the health status and utilization of healthcare services among the elderly based on socioeconomic and demographic variables, and (2) compare the utilization of healthcare services, including preventive and healthcare services, between urban and rural elderly populations in the Northeastern region of Hungary.

## Methods

### Study design and data

A cross-sectional survey was conducted from May to August 2022 to gather data from individuals aged 65 years and older residing in Northeast Hungary, which is recognized as the most deprived region in the country.

The sample was randomly selected from the population of two counties, namely Hajdú-Bihar and Szabolcs-Szatmár-Bereg. Specifically, the patients’ lists of 19 randomly selected general practitioners were considered and from each practice 25–25 randomly selected persons were invited to participate in the study. In instances where certain individuals were unavailable, alternative subjects were enrolled; however, if someone declined to participate, another person was not substituted. The intended sample size was 475 individuals (19X25 persons), out of which 443 individuals accepted the invitation, yielding a response rate of 93.3%. The survey employed a three-pillar complex health assessment, consisting of questionnaire-based interviews, physical examinations, and laboratory tests. These components were integrated to obtain comprehensive data for the study. Mortality data for the years 2017–2021 in the settlements of the study area were obtained from the Hungarian Central Statistical Office. Additionally, population data disaggregated by sex and age at the municipality level for the same time period were obtained from the Central Office for Administrative and Electronic Public Services.

The participants provided written informed consent before the start of the study. The study was conducted according to the guidelines of the Declaration of Helsinki and approved by the Ethics Committee of the Hungarian Scientific Council on Health (Reference No.: IV/3351–3/2022/EKU). All research was performed in accordance with relevant guidelines and regulations.

### Variables

The questionnaire-based interviews used questions from the Hungarian version of the European Health Interview Survey [23] to collect information on several variables, including demographic and socioeconomic factors (age, sex, educational level, marital status, living arrangements, self-reported financial status), health-related factors and health behaviour (chronic diseases, self-reported health status, limitations in activities of daily living, use of medication, smoking status, fruit and vegetable consumption), and use of health services (preventive and curative services).

Physical examinations involved obtaining anthropometric measurements (height and weight) and blood pressure (BP) readings for each participant. Body mass index (BMI) was then calculated as the ratio of weight (in kilograms) to the square of height (in meters) (kg/m^2^).

Educational attainment was categorized into three groups: primary, secondary, and tertiary. Marital status was divided into married and not married, including individuals who were single, divorced, or widowed. Living arrangement was classified based on household size as living alone (in a one-person household) or living with others. Self-reported financial status was categorized as very good/good, satisfactory, or bad/very bad.

Health-related variables included self-rated health, which was assessed using five categories ranging from very good to very bad. Activity limitation was evaluated by asking participants if health problems had limited their daily activities in the past six months and, if so, to what extent. Responses were categorized as severely limited, limited, or not limited, indicating the degree of activity limitation. Smoking status was classified as current smoker, former smoker, or non-smoker. Fruit and vegetable consumption was categorized as daily and less than daily consumption. Chronic diseases and impairments were self-reported by participants, with relevant chronic conditions considered in the analysis, including hypertension, diabetes, cardiovascular diseases, respiratory diseases, cancers, stomach or duodenal ulcer, depression, musculoskeletal disorders, and other conditions. Visual and hearing impairments were also recorded as impairments. Medication use was assessed in terms of use of prescribed medication for hypertension and diabetes.

Residential area was determined based on the location of the participants' general practitioners' (GP) practices, categorized as urban or rural. The study sample was further categorized into two age groups for multivariable analyses: 65–74 years and 75 years and older.

### Outcome measures

Health service utilization was assessed by examining both healthcare and preventive services. Healthcare services included data on visits to dentists, GPs, and medical specialists within the 12 months preceding the survey.

Preventive services utilization was evaluated in terms of general health check-ups, vaccinations, and cancer screening. General health check-ups encompassed measurements of blood pressure, blood cholesterol, and blood glucose, as well as any laboratory tests conducted within the past 12 months. Additionally, respondents were asked whether they had received an influenza vaccination in the previous 12 months and whether they had received a COVID-19 vaccination prior to the survey.

Data regarding cancer screening were obtained for colorectal, breast, and cervical cancer. The frequency of cancer screening was investigated based on the recommended time intervals for each service. This included colonoscopy within the past 5 years and faecal occult blood tests within the past 2 years, mammography within the past 2 years, and cervical cancer screening within the past 3 years preceding the survey. The sample sizes for the corresponding outcome measures varied, ranging from *n* = 267 for cervical cancer screening to *n* = 441 for COVID-19 vaccination.

### Statistical analysis

To compare the mortality rates between rural and urban settlements included in the study, the relative (rural/urban) mortality was assessed and expressed as standardised mortality ratios by age groups (25–64 years to define premature adult mortality, 65–74 years, ≥ 75 years), sex, and main causes of death. This analysis was conducted using the risk assessment module of the Rapid Inquiry Facility [24]. The expected number of cases was determined based on age- and sex-specific mortality rates for the population of the urban municipalities included in the study. Confidence intervals were obtained using statistical tables for the Poisson distribution [25]. The characteristics of the study population were described using total numbers with percentages for categorical variables and means with standard deviation for continuous variables. Descriptive statistics were performed, employing the chi-square test for categorical variables and the Mann–Whitney U test for continuous variables. For binary outcomes, multivariable logistic regression analyses were conducted to examine the effects of residential area and age on the utilization of health services. The logistic regression models were adjusted for age (in the urban–rural model), sex, education, self-reported financial status, presence of chronic diseases, and limitations in activities of daily living. The results were presented as odds ratios (OR) with their corresponding 95% confidence intervals (CI). Statistical significance was set at a p-value of less than 0.05. All analyses were performed using Stata version 13.0 software (Stata Corp., College Station, TX, USA).

## Results

Among the 443 participants in the study, 210 (47.4%) lived in rural areas, while 233 (52.6%) resided in urban areas. The proportion of men living in urban regions was lower than in rural areas (33.05% vs. 42.86%, *p* = 0.033). The majority of respondents (65.69%) were in the 65–74 years age group, and the distribution of age did not significantly differ between the two groups.

Urban residents were significantly more educated (*p* < 0.001), were more likely to live alone (*p* = 0.013), and less likely to report limitations in daily activities (*p* = 0.022), and more likely to consume fruit daily (*p* < 0.001) than rural residents. While a higher percentage of urban residents rated their health as good or very good (27.8% vs. 19.7%), there was no significant difference in self-perceived health status. Significantly higher prevalence of cancer, depression, arthrosis, osteoporosis, and elevated cholesterol level was found among urban residents, while the prevalence of heart attack was higher among those living in rural areas (Table 1). Additional data on characteristics of study population by age groups are given in Supplementary Table 1.

**Table 1:** Characteristics of the study population according to urban and rural residence

		Total sample	Rural	Urban	
		% (*n*)	% (*n*)	% (*n*)	*p*-value
		*n* = 443	*n* = 210	*n* = 233	
Sex	Male	37.7% (167)	42.86% (90)	33.05% (77)	**0.033**
	Female	62.3% (276)	57.14% (120)	66.95% (156)	
Age group	65–74 years	65.69% (291)	65.24% (137)	66.09% (154)	0.850
	75 + years	34.31% (152)	34.76% (73)	33.91% (79)	
Education level	Primary	38.32% (169)	49.28% (103)	28.45% (66)	
	Secondary	48.3% (213)	44.02% (92)	52.16% (121)	** < 0.001**
	Tertiary	13.38% (59)	6.7% (14)	19.4% (45)	
	Missing	0.45% (2)	0.48% (1)	0.43% (1)	
Living arrangement	Not alone	69.39% (306)	75.12% (157)	64.22% (149)	**0.013**
	Alone	30.61% (135)	24.88% (52)	35.78% (83)	
	Missing	0.45% (2)	0.48% (1)	0.43% (1)	
Marital status	Married	55.81% (245)	59.33% (124)	52.61% (121)	0.157
	Not married	44.19% (194)	40.67% (85)	47.39% (109)	
	Missing	0.9% (4)	0.48% (1)	1.28% (3)	
Self-perceived health	Very good	2.05% (9)	2.4% (5)	1.74% (4)	
	Good	21.92% (96)	17.31% (36)	26.09% (60)	
	Fair	60.73% (266)	65.38% (136)	56.52% (130)	0.222
	Bad	13.01% (57)	12.98% (27)	13.04% (30)	
	Very bad	2.28% (10)	1.92% (4)	2.61% (6)	
	Missing	1.12% (5)	0.95% (2)	1.3% (3)	
Limitations in activities of daily living	Not limited	42.24% (185)	36.54% (76)	47.39% (109)	**0.022**
	Limited	57.76% (253)	63.46% (132)	52.61% (121)	
	Missing	1.12% (5)	0.95% (2)	1.3% (3)	
Self-reported financial status	Very good/good	24.02% (104)	27.05% (56)	21.24% (48)	
	Satisfactory	63.51% (275)	61.84% (128)	65.04% (147)	
	Bad/very bad	12.47% (54)	11.11% (23)	13.72% (31)	0.319
	Missing	2.25% (10)	1.4% (3)	3.0% (7)	
Smoking	Never	61.99% (274)	64.76% (136)	59.48% (138)	
	Current smoker	19.00% (84)	18.57% (39)	19.4% (45)	0.430
	Former smoker	19.00% (84)	16.67% (35)	21.12% (49)	
	Missing	0.22% (1)	0% (0)	0.43% (1)	
Fruit consumption	Daily	58.77% (258)	50.00% (104)	66.67% (154)	** < 0.001**
	Less than daily	41.23% (181)	50.00% (104)	33.33% (77)	
	Missing	0.9% (4)	0.95% (2)	0.86% (2)	
Vegetable consumption	Daily	45.33% (199)	49.76% (104)	41.30% (95)	0.075
	Less than daily	54.67% (240)	50.24% (105)	58.70% (135)	
	Missing	0.9% (4)	0.48% (1)	1.29% (3)	
Physical examination	Raised blood pressure (> 130/85 mmHg)	72.01% (319)	74.29% (156)	69.96% (163)	0.311
	Body mass index (mean (SD))	29.23 (5.65)	29.28 (5.61)	29.19 (5.68)	0.566
Chronic diseases	Heart attack	4.51% (20)	7.14% (15)	2.15% (5)	**0.011**
	Coronary heart disease	18.51% (82)	15.24% (32)	21.46% (50)	0.092
	Hypertension	83.75% (371)	84.29% (177)	83.26% (194)	0.771
	Stroke	5.42% (24)	6.19% (13)	4.72% (11)	0.495
	Arrhythmia	16.03% (71)	15.24% (32)	16.74% (39)	0.667
	Asthma	9.48% (42)	7.14% (15)	11.59% (27)	0.111
	Chronic bronchitis	12.64% (56)	11.9% (25)	13.3% (31)	0.658
	Cancer	9.26% (41)	6.19% (13)	12.02% (28)	**0.035**
	Stomach or duodenal ulcer	9.93% (44)	8.57% (18)	11.16% (26)	0.363
	Depression	8.8% (39)	5.24% (11)	12.02% (28)	**0.012**
	Diabetes	25.06% (111)	24.29% (51)	25.75% (60)	0.722
	Arthrosis	34.09% (151)	27.62% (58)	39.91% (93)	**0.006**
	Osteoporosis	17.61% (78)	9.52% (20)	24.89% (58)	** < 0.001**
	Chronic kidney disease	5.64% (25)	7.62% (16)	3.86% (9)	0.087
	High cholesterol	35.89% (159)	30.48% (64)	40.77% (95)	**0.024**
Impairment	Eye disease	43.59% (187)	42.16% (86)	44.89% (101)	0.569
	Missing	3.16% (14)	2.86% (6)	3.43% (8)	
	Hearing impairment	22.83% (100)	21.63% (45)	23.91% (55)	0.571
	Missing	1.13% (5)	0.95% (2)	1.29% (3)	
Medication use	Antihypertensive medication	97.54% (357)	97.73% (172)	97.37% (185)	0.825
	Missing	1.35% (5)	0.56% (1)	2.06% (4)	
	Antidiabetic medication	100% (103)	100% (48)	100% (55)	–
	Missing	7.21% (8)	5.88% (3)	8.33% (5)	

*SD*, standard deviation. Statistically significant results (*p* < 0.05) are shown in bold

Table 2 displays the frequency of health service utilization in the study population. The highest prevalence was observed for COVID-19 vaccination (90.7%) and visits to GPs in the past year (90.2%). However, services related to cancer screening exhibited the lowest prevalence, including faecal occult blood tests (15.58%), colonoscopy (16.63%), mammography (21.98%), and cervical cancer screening (25.84%). Differences in healthcare and preventive service utilization were observed based on residential area. Rural residents, compared to urban residents, reported significantly lower frequencies of visiting dentists (20.98% vs. 32.89%; *p* = 0.006) and specialists (53.37% vs. 63.76%; *p* = 0.028), having faecal occult blood tests (8.82% vs. 21.68%; *p* < 0.001), colorectal cancer screening (12.08% vs. 20.91%; *p* = 0.014), and mammography (15.97% vs. 26.62%; *p* = 0.035). Additionally, rural residents reported a significantly higher frequency of blood pressure measurement (90.87% vs. 84.42%; *p* = 0.042). The differences in influenza vaccination and laboratory test frequency between rural and urban areas were not statistically significant (*p* = 0.073 and *p* = 0.070, respectively).

**Table 2:** Utilization of health services (including preventive and healthcare services) among elderly living in rural and urban area

	Total			Rural		Urban		*p*-value
	N	*n*	% (95% CI)	*n*	% (95% CI)	*n*	% (95% CI)	
Healthcare services								
GP visit	440	397	90.23 (87.06–92.84)	190	90.91 (86.17–94.44)	207	89.61 (84.94–93.23)	0.647
Specialist visit	437	257	58.81 (54.03–63.47)	111	53.37 (46.34–60.29)	146	63.76 (57.16–69.99)	**0.028**
Dentist visit	430	117	27.21 (23.06–31.68)	43	20.98 (15.62–27.2)	74	32.89 (26.79–39.45)	**0.006**
Vaccination								
Influenza vaccination	430	116	26.98 (22.84–31.44)	46	22.89 (17.27–29.32)	70	30.57 (24.67–36.98)	0.073
COVID-19 vaccination	441	400	90.7 (87.6–93.25)	184	88.46 (83.32–92.47)	216	92.7 (88.58–95.69)	0.126
Preventive services								
Blood pressure measurement	439	384	87.47 (84.01–90.42)	189	90.87 (86.1–94.41)	195	84.42 (79.08–88.84)	**0.042**
Cholesterol measurement	428	287	67.06 (62.38–71.49)	142	69.27 (62.46–75.51)	145	65.02 (58.37–71.27)	0.351
Blood glucose measurement	436	327	75.00 (70.66–79.00)	163	78.74 (72.54–84.11)	164	71.62 (65.3–77.36)	0.086
Faecal occult blood test	430	67	15.58 (12.28–19.36)	18	8.82 (5.31–13.59)	49	21.68 (16.49–27.63)	** < 0.001**
Colonoscopy	427	71	16.63 (13.22–20.5)	25	12.08 (7.97–17.31)	46	20.91 (15.73–26.89)	**0.014**
Mammography	273	60	21.98 (17.21–27.36)	19	15.97 (9.9–23.81)	41	26.62 (19.83–34.34)	**0.035**
Cervical cancer screening	267	69	25.84 (20.7–31.53)	26	22.22 (15.06–30.84)	43	28.67 (21.59–36.61)	0.233
Laboratory tests	429	316	73.66 (69.22–77.77)	160	77.67 (71.36–83.16)	156	69.96 (63.48–75.89)	0.070

*CI*, confidence interval; *GP*, general practitioner. Statistically significant results (*p* < 0.05) are shown in bold

Multivariable logistic regression analyses demonstrated that urban residence was significantly associated with a higher likelihood of having faecal occult blood tests (OR = 2.32, 95% CI: 1.19–4.52), and a lower likelihood of blood pressure (OR = 0.42, 95% CI: 0.21–0.86) and blood glucose (OR = 0.48, 95% CI: 0.28–0.83) measurements. While significant differences were observed between rural and urban residents in colonoscopy and mammography in the univariate analysis, these differences disappeared after adjusting for age, sex, education, self-reported financial status, chronic diseases, and limitations in daily activities. Additionally, older age (≥ 75 years) was associated with a significantly lower likelihood of GP visits (OR = 0.45, 95% CI: 0.22–0.93), mammography (OR = 0.25, 95% CI: 0.1–0.63), and cervical cancer screening (OR = 0.40, 95% CI: 0.19–0.87). The odds of receiving flu vaccination significantly increased with age (OR = 1.71, 95% CI: 1.03–2.84) (Table 3).

**Table 3:** Multivariable logistic regression model for utilization of health services (including preventive and healthcare services) among elderly

	Urban (reference: rural)			Age (reference: 65–74 years)		
	OR	95%CI	*p*-value	OR	95%CI	*p*-value
Healthcare services						
Dentist visit	1.55	(0.91–2.64)	0.107	0.72	(0.42–1.24)	0.235
GP visit	0.86	(0.40–1.85)	0.699	0.45	(0.22–0.93)	**0.031**
Specialist visit	1.28	(0.80–2.06)	0.299	0.84	(0.52–1.35)	0.478
Vaccination						
Influenza vaccination	1.45	(0.86–2.47)	0.167	1.71	(1.03–2.84)	**0.039**
COVID-19 vaccination	1.35	(0.63–2.87)	0.441	0.91	(0.42–1.95)	0.808
Preventive services						
Blood pressure measurement	0.42	(0.21–0.86)	**0.017**	0.78	(0.39–1.54)	0.470
Cholesterol measurement	0.71	(0.43–1.17)	0.177	0.79	(0.48–1.31)	0.361
Blood glucose measurement	0.48	(0.28–0.83)	**0.009**	0.89	(0.52–1.53)	0.670
Faecal occult blood test	2.32	(1.19–4.52)	**0.014**	0.59	(0.30–1.15)	0.120
Colonoscopy	1.59	(0.83–3.05)	0.159	1.00	(0.53–1.89)	0.998
Mammography	1.49	(0.69–3.22)	0.308	0.25	(0.10–0.63)	**0.003**
Cervical cancer screening	0.78	(0.38–1.59)	0.492	0.40	(0.19–0.87)	**0.020**
Laboratory tests	0.54	(0.31–0.93)	**0.028**	0.69	(0.40–1.20)	0.192

All models were adjusted for sex, education, self-reported financial status, chronic diseases, limitation in daily activities. *OR*, odds ratio; *95%CI*, 95% confidence interval; *GP*, general practitioner. Statistically significant results (*p* < 0.05) are shown in bold

Table 4 presents the relative mortality of the rural municipalities compared to the urban population. The most significant difference between urban and rural areas was observed in premature mortality (25–64 years age group). Rural areas exhibited a higher relative all-cause premature mortality than urban areas, with a 55% increase in males (SMR = 1.55, 95% CI: 1.38–1.75) and a 40% increase in females (SMR = 1.40, 95% CI: 1.16–1.69). Premature mortality in rural areas exceeded urban reference mortality by 63% for diseases of the circulatory system in males (SMR = 1.63, 95% CI: 1.31–2.01) and by 45% for malignant neoplasms (SMR = 1.45, 95% CI: 1.15–1.81) in males, and by 57% (SMR = 1.57, 95% CI: 1.04–2.29) and 39% (SMR = 1.39, 95% CI: 1.02–1.84) in females, respectively. Standardized premature mortality due to digestive diseases was significantly higher in rural areas among males (SMR = 2.30, 95% CI: 1.61–3.19), primarily due to alcoholic liver diseases (SMR = 2.57, 95% CI: 1.67–3.80). Furthermore, rural areas had more than twice the premature mortality due to COVID-19 (SMR = 2.44, 95% CI: 1.11–4.63) and colorectal cancer (SMR = 2.73, 95% CI: 1.31–5.02) in females. In adults aged 65 years and older, rural residents experienced significantly higher all-cause mortality and mortality due to circulatory diseases in both sexes. Mortality due to COVID-19 was more than twice as high in rural areas compared to urban areas in females aged 65–74 years (SMR = 2.22, 95% CI: 1.07–4.09) and in both males (SMR = 2.26, 95% CI: 1.36–3.53) and females (SMR = 2.41, 95% CI: 1.64–3.42) aged 75 years and older. Additionally, female mortality due to external causes was 2.6 times higher (SMR = 2.62, 95% CI: 1.53–4.20), mortality due to falls was 4.25 times higher (SMR = 4.25, 95% CI: 2.26–7.27), while mortality due to respiratory diseases was 47% lower among those aged 75 years and older in rural areas compared to the reference urban population.

**Table 4:** Relative mortality of the population in the rural municipalities compared to the urban population included in the study, by selected causes of death, age groups and sex, for the years 2017–2021

	Age-group		
	25–64	65–74	≥ 75
Causes of Death	Males (SMR)		
All causes of death (ICD-10: A00-Y98)	**1.55 [1.38−1.75]**	**1.27 [1.10−1.45]**	**1.23 [1.08−1.39]**
Certain infectious and parasitic diseases (ICD-10: A00-B99)	1.47 [0.04−8.21]	2.23 [0.46−6.51]	–
Diseases of the circulatory system (ICD-10: I00-I99)	**1.63 [1.31−2.01]**	**1.44 [1.15−1.77]**	**1.37 [1.16−1.62]**
Malignant neoplasms (ICD-10: C00-C97)	**1.45 [1.15−1.81]**	1.19 [0.91−1.51]	0.99 [0.72−1.34]
Malignant neoplasm of colon, rectosigmoid junction, rectum and anus (ICD-10: C18-C21)	1.00 [0.40−2.05]	1.57 [0.84−2.69]	1.55 [0.77−2.77]
Diseases of the respiratory system (ICD-10: J00-J99)	1.26 [0.60−2.32]	1.38 [0.75−2.31]	0.77 [0.42−1.30]
Diseases of the digestive system (ICD-10: K00-K93)	**2.30 [1.61−3.19]**	1.34 [0.71−2.29]	1.04 [0.38−2.26]
Alcoholic liver disease (ICD-10: K70)	**2.57 [1.67−3.80]**	1.44 [0.53−3.14]	2.17 [0.26−7.85]
External causes of morbidity and mortality (ICD-10: V01-Y98)	1.33 [0.85−2.00]	1.13 [0.41−2.45]	0.99 [0.37−2.16]
Falls (ICD-10: W00-W19)	–	0.86 [0.02−4.79]	1.06 [0.22−3.11]
Intentional self-harm (ICD-10: X60-X84)	1.94 [1.00−3.39]	1.17 [0.14−4.22]	0.57 [0.01−3.18]
COVID-19 (ICD10.: U071-U072)	1.05 [0.45−2.07]	1.05 [0.51−1.94]	**2.26 [1.36−3.53]**
	Females (SMR)		
All causes of death (ICD-10: A00-Y98)	**1.40 [1.16−1.69]**	**1.29 [1.09−1.54]**	**1.29 [1.18−1.40]**
Certain infectious and parasitic diseases (ICD-10: A00-B99)	2.71 [0.07−15.08]	1.09 [0.03−6.07]	1.39 [0.51−3.03]
Diseases of the circulatory system (ICD-10: I00-I99)	**1.57 [1.04−2.29]**	**1.76 [1.34−2.26]**	**1.43 [1.28−1.60]**
Malignant neoplasms (ICD-10: C00-C97)	**1.39 [1.02−1.84]**	0.92 [0.63−1.29]	0.96 [0.72−1.26]
Malignant neoplasm of colon, rectosigmoid junction, rectum and anus (ICD-10: C18-C21)	**2.73 [1.31−5.02]**	1.46 [0.47−3.40]	0.88 [0.38−1.74]
Malignant neoplasm of breast (ICD-10: C50)	1.66 [0.80−3.06]	1.47 [0.59−3.03]	0.62 [0.23−1.35]
Malignant neoplasm of cervix uteri (ICD-10: C53)	1.06 [0.13−3.82]	–	–
Diseases of the respiratory system (ICD-10: J00-J99)	1.42 [0.46−3.32]	1.10 [0.44−2.26]	**0.53 [0.29−0.89]**
Diseases of the digestive system (ICD-10: K00-K93)	0.47 [0.10−1.39]	1.50 [0.60−3.10]	1.02 [0.51−1.82]
Alcoholic liver disease (ICD-10: K70)	1.26 [0.26−3.69]	1.83 [0.22−6.61]	–
External causes of morbidity and mortality (ICD-10: V01-Y98)	0.59 [0.12−1.73]	0.67 [0.02−3.73]	**2.62 [1.53−4.20]**
Falls (ICD-10: W00-W19)	–	–	**4.25 [2.26−7.27]**
Intentional self-harm (ICD-10: X60-X84)	–	2.84 [0.07−15.84]	1.20 [0.03−6.70]
COVID-19 (ICD10.: U071-U072)	**2.44 [1.11−4.63]**	**2.22 [1.07−4.09]**	**2.41 [1.64−3.42]**
	Both (SMR)		
All causes of death (ICD-10: A00-Y98)	**1.50 [1.36−1.66]**	**1.28 [1.15−1.42]**	**1.27 [1.18−1.36]**
Certain infectious and parasitic diseases (ICD-10: A00-B99)	1.91 [0.23−6.89]	1.77 [0.48−4.52]	0.98 [0.36−2.13]
Diseases of the circulatory system (ICD-10: I00-I99)	**1.62 [1.35−1.94]**	**1.55 [1.32−1.83]**	**1.41 [1.29−1.55]**
Malignant neoplasms (ICD-10: C00-C97)	**1.43 [1.20−1.70]**	1.08 [0.88−1.32]	0.98 [0.79−1.20]
Malignant neoplasm of colon, rectosigmoid junction, rectum and anus (ICD-10: C18-C21)	1.59 [0.93−2.55]	1.54 [0.91−2.43]	1.17 [0.71−1.83]
Diseases of the respiratory system (ICD-10: J00-J99)	1.31 [0.73−2.16]	1.27 [0.78−1.94]	**0.63 [0.42−0.91]**
Diseases of the digestive system (ICD-10: K00-K93)	**1.78 [1.26−2.43]**	1.39 [0.85−2.15]	1.02 [0.60−1.64]
Alcoholic liver disease (ICD-10: K70)	**2.32 [1.54−3.35]**	1.52 [0.66−3.00]	1.25 [0.15−4.53]
External causes of morbidity and mortality (ICD-10: V01-Y98)	1.17 [0.76−1.71]	1.03 [0.41−2.11]	**1.84 [1.16−2.76]**
Falls (ICD-10: W00-W19)	**–**	0.68 [0.02−3.76]	**2.72 [1.56−4.42]**
Intentional self-harm (ICD-10: X60-X84)	1.46 [0.75−2.55]	1.45 [0.30−4.25]	0.77 [0.09−2.79]
COVID-19 (ICD10.: U071-U072)	1.5 [0.88−2.41]	1.43 [0.87−2.21]	**2.35 [1.74−3.1]**

*SMR*, standardised mortality ratio; *ICD*, international classification of diseases. Statistically significant results (*p* < 0.05) are shown in bold

## Discussion

This study provides valuable insights into the health status and utilization of preventive health services among older adults living in rural and urban areas of Northeast Hungary, addressing a current data gap in CEE countries. Consistent with findings from other CEE countries [9, 10, 26, 27], our study reveals generally poor health status among older adults in Hungary. A significant proportion of participants rated their health as fair, poor, or very poor, with no significant difference in self-perceived health status between urban and rural areas, although urban residents reported slightly better health status. The role of social network and social participation in influencing self-rated health has been observed in urban residents, while loneliness is associated with declining self-rated health in rural residents [26]. Moreover, limitations in daily activities were more prevalent among rural residents, aligning with previous research [28]. Urban residents exhibited a higher prevalence of chronic diseases [26, 29], which may be attributed to better access to healthcare facilities, accurate diagnosis, and a higher likelihood of seeking medical care.

Our study highlights low participation rates in preventive health services such as dentist visits, influenza vaccination, and cancer screening programs, while GP visits, cardiometabolic preventive services, and COVID-19 vaccination showed relatively high utilization rates. Significant disparities were observed in the utilization of health services between rural and urban areas. However, after adjusting for potential confounders, only certain cardiometabolic preventive services, laboratory tests, and colorectal cancer screening by faecal occult blood test demonstrated significant differences between rural and urban residents.

Controlling cardiometabolic risk factors is crucial in reducing the risk of cardiovascular diseases [30], improving life expectancy [31], and mitigating physical limitations [32] in older age. Our study reveals a relatively high utilization rate of cardiometabolic preventive services, particularly among rural residents. The presence of a higher density of GPs in urban areas may result in lower opportunity costs for care, including travel expenses and waiting time [33], thereby increasing the likelihood of utilizing preventive services [34]. Notably, a high proportion of participants visited a GP within the past year, which may explain the higher utilization of services for the prevention of cardiometabolic diseases among them.

Participation in breast and cervical cancer screening is recommended for women under 65 years in Hungary. However, since a significant proportion of cases is diagnosed in older ages, continued screening would result in increased life expectancy [35–37]. Our study found low participation rates in mammography and cervical cancer screening among older adults, which were considerably lower than reported by the OECD in the target age group [38]. Similarly low participation rates were observed for colorectal cancer screenings. Although breast and colon cancer screening services were significantly lower among rural residents, adjusting for demographic, socioeconomic, and health status variables eliminated the significance, except for faecal occult blood testing. Limited availability of cancer screening technology and human resource shortages, particularly in less developed regions, remain significant challenges in cancer care in Hungary [38, 39].

No significant differences were found in COVID-19 and influenza vaccination rates between rural and urban residents. Although influenza vaccination is free for the population aged 65 years and over in Hungary, vaccination coverage (27%) was far from the recommended level (75% according to WHO recommendation). Even though influenza vaccination is a cost-effective way to reduce premature mortality, the vaccination rate against influenza among older people is low in CEE countries [6], which could be explained by socio-demographic factors, health status and health behaviour [40], and the lack of perceived need and recommendation by their physician for vaccine [6]. COVID-19 vaccination rate was at around 90% in the study population. Although the proportion of those vaccinated against COVID-19 did not differ between rural and urban residents, our analysis showed significantly higher mortality rates due to COVID-19 among females in all age groups and among males aged 75 years and older in rural areas. Rural areas generally have an older population with a higher prevalence of pre-existing conditions and comorbidities, which are known risk factors for severe COVID-19 illness and death including cardiovascular disease (CVD), hypertension, diabetes and obesity [41–43]. In addition, a recent study highlighted that the dysfunction of the heart-brain axis during SARS‐CoV‐2 infection may worsen the outcome of the COVID-19 patients through neurotropism or by increasing the susceptibility to psychosocial factors that may lead to neurological and stress-related diseases associated with CVD [44]. Although, the outcome of heart and brain disease in COVID-19 patients depends on their sex [45]. Furthermore, previous research conducted in Hungary has revealed that residents of more deprived municipalities face a higher risk of COVID-19 mortality, even though they are less likely to be identified as confirmed COVID-19 cases [46]. These findings underscore the presence of significant regional disparities in healthcare access and outcomes.

Previous studies have highlighted the health inequalities that exist between rural and urban populations. A cross-sectional study, focusing on 11 high-income countries investigated health disparities across 10 indicators and found differences between rural and urban areas, particularly in access to primary care [47]. Nevertheless, our study showed no difference in the frequency of GP’s visits between urban and rural residents. Others found a higher prevalence of smoking among those living in urban areas [48], which was not supported by our results. However, it is worth noting that the differences in health status and service utilization between rural and urban elderly populations were less pronounced than expected, which may be attributed to the higher premature mortality observed in rural areas. Our data indicate substantial rural–urban disparities in all-cause mortality and mortality from major conditions in the municipalities covered by this study. Rural residents, particularly males, exhibited higher premature mortality rates due to cardiovascular diseases, cancers, and diseases of the digestive system (particularly for alcoholic liver disease), while rural females experienced higher mortality rates due to COVID-19, falls, and external causes. These findings align with previous studies from other countries where rural areas demonstrated higher all-cause mortality, cardiovascular disease mortality and cancer mortality [49, 50], along with lower respiratory disease mortality [51]. However, these differences in mortality could be partly explained by the deprivation level of the area [46, 51]. Furthermore, similar to our findings, a shift from urban to rural excess mortality with age can be observed in several European countries [52]. It is important to note that the high premature mortality rate among rural residents may result in the exclusion of the most vulnerable population groups from our study, leading to a relatively healthier elderly rural population with higher levels of health literacy and positive attitudes towards health services. The presence of such marked rural–urban disparities in mortality patterns underscores the need to address health inequalities and implement interventions to prevent premature mortality through lifestyle changes and improved access to medical services.

Individuals' beliefs and attitudes toward aging can impact the utilization of health services among older adults. Positive self-perception of aging has been linked to better preventive health behaviour [53] and a higher likelihood of using various preventive services [54]. On the other hand, older adults who believe that health problems are inevitable in old age are less likely to have regular physician visits and use preventive services [55]. Factors such as cognitive impairment [56] also influence the utilization of health services among the elderly. Besides beliefs and attitude of older adults, health care system factors can also influence the use of different services. Common reasons reported by older adults for not obtaining the needed medical care include cost, long waiting time for an appointment and lack of transportation or distance [19]. Generally, the number of physicians per capita is lower in rural areas, resulting in the concentration of specialized services in urban areas [10]. In addition to the limited range of available services, there are longer travel distances and limited transport options, thus existing disabilities particularly in older ages may further limit access to services. In Hungary, the differences in the density of doctors between urban and rural areas are particularly large [10], and health workforce shortage is a major concern in the country [57]. Age itself is also considered as an important determinant of utilisation of health services [34]. In our study the probability of visiting a GP, and receiving mammography and cervical cancer screening significantly decreased with age, which underline that access to certain medical services may be limited due to age. Furthermore, the findings suggest that the importance of participation in screening program by women above the recommended age limit (over 65 years) should be better communicated to the older population.

Preventive services offer a cost-effective approach to prevent and reduce the burden of chronic diseases and associated disabilities among the elderly. Increasing the utilization of specific preventive services and addressing the underutilized services, such as colorectal cancer screening and influenza vaccinations, can contribute to population health improvement, extended healthy life years, and enhanced quality of life for older adults [58]. With healthy lifestyle and proper medical care it becomes possible even in older ages to prevent or postpone the occurrence and development of certain risk factors and conditions and to reduce disability and dependency due to chronic diseases, which may have a long-lasting impact on older adult's quality of life [59]. The demographic shift will require increased attention from health policy to health service provision for older people, since an increased focus on avoidable conditions and enhanced preventive health services is essential to achieve a healthy ageing population [60]. Health care systems have to address the needs of ageing populations, implement policies that promote lifelong health and emphasize preventive care to prevent or delay the onset of age-related disability and ensure the well-being [2].

Rurality does not inevitably lead to urban–rural disparities, although it may exacerbate the impact of certain determinants including poor access to health services (due to travel distances and limited transport), socioeconomic deprivation and personal risk factors on health disparities between rural and urban residents [61]. Therefore, a comprehensive health care policy is needed that includes interventions that target health literacy and health-seeking behaviour, as well as ensuring the availability of health services [61]. Since access to health services is an important determinant of health outcomes for both curative and preventive care, there is a need to improve the accessibility of services in terms of adequate transport, particularly for older people with activity limitations.

Several limitations of this study should be acknowledged. Firstly, the use of self-reported measures for health status and service utilization may be susceptible to recall bias, potentially leading to overreporting of service utilization and prevalence of chronic diseases. Secondly, although we adjusted for major socioeconomic and health-related factors in our multivariable models, there may be other independent variables (e.g., beliefs, attitudes) that could influence the results but were not included. Thirdly, the underlying reasons for medical care visits and the use of health services were not captured in our data, limiting our understanding of the motivations behind service utilization. Fourthly, our study focuses on a specific region of Hungary, which may limit the generalizability of the findings to the entire country or other CEE countries. Lastly, as our data collection took place during the COVID-19 pandemic, the pandemic's impact on the outcome variables cannot be ignored.

In conclusion, our study highlights that the utilization of health services among older adults is associated with their place of residence. Although utilization of services provided by specialists, particularly cancer screening, is more frequent among urban residents, a higher utilization of preventive services for cardiometabolic risk factors is observed among rural residents. However, the high premature mortality in rural areas may moderate the health differences between rural and urban elderly populations. These findings emphasize the importance of preventive measures in older age and the need to reduce inequalities in mortality between rural and urban areas. Addressing barriers to medical care among older adults has the potential to increase the utilization of preventive services, contributing to reduced mortality, increased healthy life expectancy, and improved quality of life. Our findings suggest that geographical regions may have different capacities to address the increasing health problems of older age groups; therefore, it would be beneficial to analyse urban–rural disparities in a more diverse range of regions and countries. Comprehensive studies are needed to further describe the health needs of older populations and implement policies that promote healthy aging in CEE countries.

### Supplementary Information

Below is the link to the electronic supplementary material.

Supplementary file1 (DOCX 24 KB)

## Data Availability

The data are available from the corresponding author upon reasonable request due to privacy or ethical concerns.

## References

[1] Medici AC. Health sector challenges and policies in the context of ageing populations. United Nations, Department of Economics and Social Affairs, Population Division, UN DESA/POP/2021/TP/NO. 3; 2021. Available from: https://desapublications.un.org/working-papers/health-sector-challenges-and-policies-context-ageing-populations. Accessed 9 June 2023.

[2] United Nations, Department of Economic and Social Affairs, Population Division. World Population Prospects: The 2015 Revision, Key Findings and Advance Tables. Working Paper No. ESA/P/WP.241. 2015. Available from: https://www.un.org/en/development/desa/publications/world-population-prospects-2015-revision.html. Accessed 8 June 2023.

[3] He W, Goodkind D, Kowal P. An Aging World: 2015. U.S. Census Bureau; 2016. Report No.: International Population Reports, P95/16–1. Available from: https://www.census.gov/library/publications/2016/demo/P95-16-1.html. Accessed 8 June 2023.

[4] European Commission. The 2018 Ageing Report – Economic and budgetary projections for the EU Member States (2016–2070). 2018. Available from: https://economy-finance.ec.europa.eu/publications/2018-ageing-report-economic-and-budgetary-projections-eu-member-states-2016-2070_en. Accessed 4 June 2023.

[5] Kurpas D, Stefanicka-Wojtas D, Shpakou A, Halata D, Mohos A, Skarbaliene A (2021). The advantages and disadvantages of integrated care implementation in Central and Eastern Europe – perspective from 9 CEE countries. Int J Integr Care.

[6] OECD. Health at a Glance: Europe 2020: State of Health in the EU Cycle. Paris: Organisation for Economic Co-operation and Development; 2020 [cited 2023 Jun 7]. Available from: https://www.oecd-ilibrary.org/social-issues-migration-health/health-at-a-glance-europe-2020_82129230-en

[7] Bíró A, Branyiczki R (2020). Transition shocks during adulthood and health a few decades later in post-socialist Central and Eastern Europe. BMC Public Health.

[8] Knurowski T, Lazić D, van Dijk JP, Geckova AM, Tobiasz-Adamczyk B, van den Heuvel WJA (2004). Survey of health status and quality of life of the elderly in Poland and Croatia. Croat Med J.

[9] Ghinescu M, Olaroiu M, van Dijk JP, Olteanu T, van den Heuvel WJA (2014). Health status of independently living older adults in Romania. Geriatr Gerontol Int.

[10] OECD. Health at a Glance 2021: OECD Indicators. Paris: Organisation for Economic Co-operation and Development; 2021 [cited 2023 Jun 7]. Available from: https://www.oecd-ilibrary.org/social-issues-migration-health/health-at-a-glance-2021_ae3016b9-en

[11] OECD. Health at a Glance: Europe 2022: State of Health in the EU Cycle. Paris: Organisation for Economic Co-operation and Development; 2022 [cited 2023 Jun 8]. Available from: https://www.oecd-ilibrary.org/social-issues-migration-health/health-at-a-glance-europe-2022_507433b0-en

[12] OECD. Potential years of life lost (indicator). 2023 [cited 2023 Jun 8]. Available from: 10.1787/193a2829-en

[13] Hungarian Central Statistical Office. [cited 2023 Jun 8]. Available from: https://www.ksh.hu/

[14] Chen C, Goldman DP, Zissimopoulos J, Rowe JW, Research Network on an Aging Society (2018). Multidimensional comparison of countries’ adaptation to societal aging. Proc Natl Acad Sci USA.

[15] Uzzoli A (2016). Health inequalities regarding territorial differences in Hungary by discussing life expectancy. Reg Stat.

[16] Juhász A, Nagy C, Páldy A, Beale L (2010). Development of a Deprivation Index and its relation to premature mortality due to diseases of the circulatory system in Hungary, 1998–2004. Soc Sci Med.

[17] Nagy C, Juhász A, Beale L, Páldy A (2012). Mortality amenable to health care and its relation to socio-economic status in Hungary, 2004–08. Eur J Pub Health.

[18] Marmot M, Friel S, Bell R, Houweling TAJ, Taylor S, Commission on Social Determinants of Health (2008). Closing the gap in a generation: health equity through action on the social determinants of health. Lancet..

[19] Okoro CA, Strine TW, Young SL, Balluz LS, Mokdad AH (2005). Access to health care among older adults and receipt of preventive services. Results from the behavioral risk factor surveillance system, 2002. Prev Med.

[20] Chawla M, Betcherman G, Banerji A. From Red to Gray : The ‘Third Transition’ of Aging Populations in Eastern Europe and the Former Soviet Union . Washington, DC: World Bank; 2007 [cited 2023 Jun 7]. Available from: http://hdl.handle.net/10986/6741

[21] Wilson A, Windak A, Oleszczyk M, Wilm S, Hasvold T, Kringos D. The delivery of primary care services. Building primary care in a changing Europe. European Observatory on Health Systems and Policies; 2015 [cited 2023 Jun 8]. Available from: https://www.ncbi.nlm.nih.gov/books/NBK458725/

[22] Ono T, Schoenstein M, Buchan J. Geographic Imbalances in Doctor Supply and Policy Responses. Paris: OECD; 2014. Available from: https://www.oecd-ilibrary.org/social-issues-migration-health/geographic-imbalances-in-doctor-supply-and-policy-responses_5jz5sq5ls1wl-en

[23] Hungarian Central Statistical Office. Health Interview Survey (EHIS), 2014. Questionnaire. 2014. Available from: https://www.ksh.hu/elef/archiv/2014/pdfs/elef2014_kerdoiv.pdf. Accessed 5 June 2023.

[24] Beale L, Hodgson S, Abellan JJ, Lefevre S, Jarup L (2010). Evaluation of spatial relationships between health and the environment: the rapid inquiry facility. Environ Health Perspect.

[25] Dobson AJ, Kuulasmaa K, Eberle E, Scherer J (1991). Confidence intervals for weighted sums of Poisson parameters. Stat Med.

[26] Tobiasz-Adamczyk B, Zawisza K (2017). Urban-rural differences in social capital in relation to self-rated health and subjective well-being in older residents of six regions in Poland. Ann Agric Environ Med.

[27] Lee S (2021). Social exclusion and subjective well-being among older adults in Europe: findings from the European social survey. J Gerontol: B.

[28] Moon J-H (2019). Factors affecting activity limitation in the elderly: data processed from the Korea National Health and Nutrition Examination Survey, 2016. Osong Public Health Res Perspect.

[29] Chauhan S, Srivastava S, Kumar P, Patel R (2022). Decomposing urban-rural differences in multimorbidity among older adults in India: a study based on LASI data. BMC Public Health.

[30] Yusuf S, Hawken S, Ôunpuu S, Dans T, Avezum A, Lanas F (2004). Effect of potentially modifiable risk factors associated with myocardial infarction in 52 countries (the INTERHEART study): case-control study. Lancet.

[31] Clarke R, Emberson J, Fletcher A, Breeze E, Marmot M, Shipley MJ (2009). Life expectancy in relation to cardiovascular risk factors: 38 year follow-up of 19 000 men in the Whitehall study. BMJ.

[32] Welmer A-K, Angleman S, Rydwik E, Fratiglioni L, Qiu C (2013). Association of cardiovascular burden with mobility limitation among elderly people: a population-based study. PLoS One.

[33] Jusot F, Or Z, Sirven N (2011). Variations in preventive care utilisation in Europe. Eur J Ageing.

[34] Agrawal S, Makuch S, Lachowicz G, Dróżdż M, Dudek K, Mazur G (2021). How sociodemographic factors impact the utilization of recommended clinical preventive screening services in Poland: a nationwide cross-sectional study. Int J Environ Res Public Health.

[35] Lichter KE, Levinson K, Hammer A, Lippitt MH, Rositch AF (2022). Understanding cervical cancer after the age of routine screening: characteristics of cases, treatment, and survival in the United States. Gynecol Oncol.

[36] Yost S, Hoekstra A (2018). Cervical cancer in women over 65: An analysis of screening. Gynecol Oncol Rep.

[37] Braithwaite D, Demb J, Henderson LM (2016). Optimal breast cancer screening strategies for older women: current perspectives. Clin Interv Aging.

[38] OECD. EU Country Cancer Profile: Hungary 2023. Paris: Organisation for Economic Co-operation and Development; 2023 [cited 2023 Jun 7]. Available from: https://www.oecd-ilibrary.org/social-issues-migration-health/eu-country-cancer-profile-hungary-2023_ccaf0398-en

[39] Scholz N. Addressing Health Inequalities in the European Union-Concepts, Action, State of Play. European Union; 2020.

[40] Schmitz H, Wübker A (2011). What determines influenza vaccination take-up of elderly Europeans?. Health Econ.

[41] Peters DJ (2020). Community susceptibility and resiliency to COVID-19 across the rural-urban continuum in the United States. J Rural Health.

[42] Wu Z, McGoogan JM (2020). Characteristics of and important lessons from the Coronavirus Disease 2019 (COVID-19) outbreak in China: summary of a report of 72 314 cases from the Chinese center for disease control and prevention. JAMA.

[43] National Center for Immunization and Respiratory Diseases (NCIRD), Division of Viral Diseases. Science Brief: Evidence Used to Update the List of Underlying Medical Conditions Associated with Higher Risk for Severe COVID-19. CDC COVID-19 Science Briefs . Atlanta (GA): Centers for Disease Control and Prevention (US); 2020 [cited 2023 Jun 27]. Available from: https://www.cdc.gov/coronavirus/2019-ncov/hcp/clinical-care/underlyingconditions.html 34009770

[44] Lionetti V, Bollini S, Coppini R, Gerbino A, Ghigo A, Iaccarino G (2021). Understanding the heart-brain axis response in COVID-19 patients: a suggestive perspective for therapeutic development. Pharmacol Res.

[45] Baroni C, Lionetti V (2021). The impact of sex and gender on heart-brain axis dysfunction: current concepts and novel perspectives. Can J Physiol Pharmacol.

[46] Oroszi B, Juhász A, Nagy C, Horváth JK, McKee M, Ádány R (2021). Unequal burden of COVID-19 in Hungary: a geographical and socioeconomic analysis of the second wave of the pandemic. BMJ Glob Health.

[47] MacKinnon NJ, Emery V, Waller J, Ange B, Ambade P, Gunja M (2023). Mapping health disparities in 11 high-income nations. JAMA Netw Open.

[48] Völzke H, Neuhauser H, Moebus S, Baumert J, Berger K, Stang A (2006). Urban-rural disparities in smoking behaviour in Germany. BMC Public Health.

[49] Singh GK, Siahpush M (2014). Widening rural-urban disparities in all-cause mortality and mortality from major causes of death in the USA, 1969–2009. J Urban Health.

[50] Lagacé C, Desmeules M, Pong RW, Heng D (2007). Non-communicable disease and injury-related mortality in rural and urban places of residence: a comparison between Canada and Australia. Can J Public Health.

[51] Gartner A, Farewell D, Roach P, Dunstan F (2011). Rural/urban mortality differences in England and Wales and the effect of deprivation adjustment. Soc Sci Med.

[52] Ebeling M, Rau R, Sander N, Kibele E, Klüsener S (2022). Urban–rural disparities in old-age mortality vary systematically with age: evidence from Germany and England & Wales. Public Health.

[53] Levy BR, Myers LM (2004). Preventive health behaviors influenced by self-perceptions of aging. Prev Med.

[54] Kim ES, Moored KD, Giasson HL, Smith J (2014). Satisfaction with aging and use of preventive health services. Prev Med.

[55] Goodwin JS, Black SA, Satish S (1999). Aging versus disease: the opinions of older black, Hispanic, and non-Hispanic white Americans about the causes and treatment of common medical conditions. J Am Geriatr Soc.

[56] Kang S, Xiang X (2022). Cognitive impairment as a barrier to utilizing preventive health services among older adults. Arch Gerontol Geriatr.

[57] Papp M, Kőrösi L, Sándor J, Nagy C, Juhász A, Ádány R (2019). Workforce crisis in primary healthcare worldwide: Hungarian example in a longitudinal follow-up study. BMJ Open.

[58] Maciosek MV, LaFrance AB, Dehmer SP, McGree DA, Flottemesch TJ, Xu Z (2017). Updated priorities among effective clinical preventive services. Ann Fam Med.

[59] Goldberg TH, Chavin SI (1997). Preventive medicine and screening in older adults. J Am Geriatr Soc.

[60] Oxley H. Policies for healthy ageing: an Overview. Paris: OECD; 2009. Available from: https://www.oecd-ilibrary.org/social-issues-migration-health/policies-for-healthy-ageing_226757488706

[61] Smith KB, Humphreys JS, Wilson MGA (2008). Addressing the health disadvantage of rural populations: how does epidemiological evidence inform rural health policies and research?. Aust J Rural Health.

