# Supplementary material for: Comparative analysis of health status and health service utilization patterns among rural and urban elderly populations in Hungary: a study on the challenges of unhealthy aging
Source: GeroScience. 2023 Oct 6;46(2):2017–31. doi: 10.1007/s11357-023-00926-y (PMC10828334; doi:10.1007/s11357-023-00926-y)
Supplement: Supplementary file 1 — Supplementary file1 (DOCX 24 KB) [file 11357_2023_926_MOESM1_ESM.docx]

Comparative Analysis of Health Status and Health Service Utilization Patterns among Rural and Urban Elderly Populations in Hungary: A Study on the Challenges of Unhealthy Aging

Nora Kovacs^1,2^, Peter Piko^1,3^, Attila Juhasz^3^, Csilla Nagy^3^, Beatrix Oroszi^3^, Zoltan Ungvari^4,5,6,7^, Roza Adany^1,2,3,8^

^1^ Department of Public Health and Epidemiology, Faculty of Medicine, University of Debrecen, Debrecen, Hungary

^2^ ELKH-DE Public Health Research Group, Department of Public Health and Epidemiology, Faculty of Medicine, University of Debrecen, Debrecen, Hungary

^3^ National Laboratory for Health Security, Center for Epidemiology and Surveillance, Semmelweis University, Budapest, Hungary

^4^ Vascular Cognitive Impairment and Neurodegeneration Program, Oklahoma Center for Geroscience and Healthy Brain Aging, Department of Biochemistry and Molecular Biology, University of Oklahoma Health Sciences Center, Oklahoma City, USA.

^5^ Department of Health Promotion Sciences, College of Public Health, University of Oklahoma Health Sciences Center, Oklahoma City, USA.

^6^ International Training Program in Geroscience, Doctoral School of Basic and Translational Medicine/Departments of Public Health and Translational Medicine, Semmelweis University, Budapest, Hungary.

^7^ The Peggy and Charles Stephenson Cancer Center, University of Oklahoma Health Sciences Center, Oklahoma City, USA.

^8^ Department of Public Health, Semmelweis University, Budapest, Hungary

Corresponding author:

Roza Adany, MD, PhD

Department of Public Health, Semmelweis University, Budapest, Hungary

[adany.roza@semmelweis.hu](mailto:adany.roza@semmelweis.hu) or adany.roza@med.unideb.hu

Supplementary Table 1 Characteristics of the study population by age groups

|  |  | 65-74 years | 75+ years |  |
| --- | --- | --- | --- | --- |
|  |  | % (n) | % (n) | p-value |
|  |  | n=291 | n=152 |  |
| Sex | male | 38.83% (113) | 35.53% (54) | 0.496 |
|  | female | 61.17% (178) | 64.47% (98) |  |
| Education level | primary | 31.38% (91) | 51.66% (78) | **<0.001** |
|  | secondary | 53.45% (155) | 38.41% (58) |  |
|  | tertiary | 15.17% (44) | 9.93% (15) |  |
|  | missing | 0.34% (1) | 0.65% (1) |  |
| Living arrangement | not alone | 76.21% (221) | 56.29% (85) | **<0.001** |
|  | alone | 23.79% (69) | 43.71% (66) |  |
|  | missing | 0.34% (1) | 0.65% (1) |  |
| Marital status | married | 62.85% (181) | 42.38% (64) | **<0.001** |
|  | not married | 37.15% (107) | 57.62% (87) |  |
|  | missing | 1.03% (3) | 0.65% (1) |  |
| Self-perceived health | very good | 2.43% (7) | 1.33% (2) | **<0.001** |
|  | good | 26.74% (77) | 12.67% (19) |  |
|  | fair | 60.07% (173) | 62% (93) |  |
|  | bad | 8.68% (25) | 21.33% (32) |  |
|  | very bad | 2.08% (6) | 2.67% (4) |  |
|  | missing | 1.03% (3) | 1.31% (2) |  |
| Limitations in activities of daily living | no limitation | 47.92% (138) | 31.33% (47) | **0.001** |
|  | limitation | 52.08% (150) | 68.67% (103) |  |
|  | missing | 1.03% (3) | 1.31% (2) |  |
| Self-reported financial status | very good/good | 24.21% (69) | 23.65% (35) | 0.982 |
|  | satisfactory | 63.51% (181) | 63.51% (94) |  |
|  | bad/very bad | 12.28% (35) | 12.84% (19) |  |
|  | missing | 2.06% (6) | 2.63% (4) |  |
| Smoking | never | 54.48% (158) | 76.16% (115) | **<0.001** |
|  | current smoker | 23.79% (69) | 9.93% (15) |  |
|  | former smoker | 21.72% (63) | 13.91% (21) |  |
|  | missing | 0.34% (1) | 0% (0) |  |
| Fruit consumption | daily | 59.72% (172) | 56.95% (86) | 0.576 |
|  | less than daily | 40.28% (116) | 43.05% (65) |  |
|  | missing | 1.03% (3) | 0.65% (1) |  |
| Vegetable consumption | daily | 47.92% (138) | 40.40% (61) | 0.133 |
|  | less than daily | 52.08% (150) | 59.60% (90) |  |
|  | missing | 1.03% (3) | 0.65% (1) |  |
| Physical examination | Raised blood pressure (>130/85) | 70.45% (205) | 75% (114) | 0.311 |
|  | BMI (mean (SD)) | 29.39 (5.83) | 28.94 (5.27) | 0.498 |
|  | missing | 0.69% (2) | 0% (0) |  |
| Chronic diseases | heart attack | 3.78% (11) | 5.92% (9) | 0.303 |
|  | coronary heart disease | 17.53% (51) | 20.39% (31) | 0.460 |
|  | hypertension | 79.73% (232) | 91.45% (139) | **0.001** |
|  | stroke | 5.15% (15) | 5.92% (9) | 0.735 |
|  | arrhythmia | 17.18% (50) | 13.82% (21) | 0.359 |
|  | asthma | 10.65% (31) | 7.24% (11) | 0.244 |
|  | chronic bronchitis | 12.71% (37) | 12.5% (19) | 0.949 |
|  | cancer | 8.93% (26) | 9.87% (15) | 0.747 |
|  | stomach or duodenal ulcer | 9.97% (29) | 9.87% (15) | 0.974 |
|  | depression | 6.87% (20) | 12.5% (19) | **0.047** |
|  | diabetes | 23.02% (67) | 28.95% (44) | 0.172 |
|  | arthrosis | 29.55% (86) | 42.76% (65) | **0.005** |
|  | osteoporosis | 17.53% (51) | 17.76% (27) | 0.950 |
|  | chronic kidney disease | 5.15% (15) | 6.58% (10) | 0.537 |
|  | high cholesterol | 38.83% (113) | 30.26% (46) | 0.074 |
| Impairment | eye disease | 43.26% (122) | 44.22% (65) | 0.85 |
|  | missing | 3.09% (9) | 3.29% (5) |  |
|  | hearing impairment | 19.86% (57) | 28.48% (43) | **0.041** |
|  | missing | 1.37% (4) | 0.65% (1) |  |
|  | Antihypertensive medication | 97.81% (223) | 97.10% (134) | 0.673 |
| Medication use | missing | 1.72% (4) | 0.72% (1) |  |
|  | Antidiabetic medication | 100.00% (63) | 100.00% (40) | - |
|  | missing | 5.97% (4) | 9.09% (4) |  |

SD, standard deviation. Statistically significant results (p<0.05) are shown in bold.
